# Evaluation of Combined Use of Protein and Postbiotics Feed Supplements in Honey Beehives in Autumn and Spring

**DOI:** 10.1007/s12602-025-10489-3

**Published:** 2025-02-24

**Authors:** Eduardo J. García-Vicente, María Benito-Murcia, María Martín, Ana Pérez, Noelia Hermosilla, Juan Manuel Alonso, David Risco

**Affiliations:** 1 Department of Animal Medicine, Faculty of Veterinary, Universidad de Extremadura. Av. de la Universidad S/N, 10003 Cáceres, Extremadura Spain; 2 Neobéitar S.L. Av. de Alemania, 6 1ºB, 10001 Cáceres, Extremadura Spain; 3 Department of Animal Health, Faculty of Veterinary, Universidad de Extremadura. Av. de la Universidad S/N, 10003 Cáceres, Extremadura Spain

**Keywords:** Honey bees, Supplementary feed, Protein, Postbiotics, Strength, Sanitary status

## Abstract

Feed supplementation in beehives is a commonly necessary practice in beekeeping today, to address the many risks the honey bees face, mainly the climate change and times of food scarcity, agricultural pesticides, or pathogens such as *Varroa destructor* or *Nosema ceranae*. Protein supplements and postbiotics products have been evaluated in beehives feed, obtaining improvements of the strength and sanitary status, but they have not been tested in combination. In this study, two experiences have been carried out in autumn and spring to check the potential beneficial effect of this combination in the beehives. Two monitoring were carried out, before and after 1 month of the application of products, in order to assess the strength (number of bees, amount of brood, brood survival, and pollen/honey reserves) and sanitary status (*V. destructor* and *N. ceranae* infestation levels) of the colonies. Firstly, the results obtained in autumn experience showed a reduction of both pathogens in supplemented group. On the other hand, in spring, the combination of protein and postbiotics enhanced the brood survival and honey yield, and decreased the *V. destructor* infestation. Thus, the combined use of protein and postbiotics as feed supplements could be an important tool to improve the sanitary status after the cold season, and to increase the strength and production of beehives in spring, unifying the benefits of both supplements.

## Introduction

Beekeeping is an essential activity in many regions of the world with an economic, ecological, and cultural primordial role [1–4]; however, it faces many risks that jeopardize its continuity. In last decades, beehives’ mortality rates of up to 40% have been registered, specifically in the winter period, due to a multifactorial etiology [5–8]. Among them, some such as the climate change, the intensive use of pesticides, or some pathogenic agents such as *Varroa destructor* or *Nosema ceranae* stand out. The mechanisms by which these factors can compromise the viability and survival of the beehives are, among others, the deterioration of the nutritional status of the beehives, or the predisposition to harmful agents [9–14].

On the one hand, the climate change is increasing the mean temperatures and causing changes in the normal climate conditions [15]. Specifically, the Iberian Peninsula experiences extreme temperatures in summer, and abnormal weather conditions that alter the typical flowering patterns [16]. This may provoke long periods of food scarcity for the bees, leading to a malnutrition situation [9, 17]. On the other hand, *V. destructor* is a parasitic mite that is worldwide distributed and feeds on the fat body and hemolymph of the bees. Thus, a high infestation by this mite can reduce the corporal reserves of the bees, longevity, and functionality. In fact, climate change and *V. destructor* are intimately related [18–21]. Another consequence of climate change in this region is fewer cold winters, without cessation of queen laying during the cold season. This makes the infestation by *V. destructor* management more difficult, since the constant presence of brood allows greater reproduction of the mites, and decreases the efficiency of treatments [16, 22, 23].

This scenario is leading to the necessary use of supplementary feeding to ensure a good nutritional status of the beehives and its survival through the cold season. Some of the key points to realize these supplementations are pre-winter period, the beginning of spring, or summer. Firstly, in autumn, a correct entry of food into the beehive, specifically protein, is essential to ensure the viability and longevity of bees born at this time, which must survive through winter [24, 25]. Furthermore, feed supplementation at the beginning of the spring is a normal practice in many regions, because of the need to generate a good strength in the beehive with a large amount of brood and a high number of forager bees that can take advantage of a target flowering [26, 27]. Finally, summers are becoming hotter and involve long periods of scarcity of nectar and pollen in the field [28].

Another question that needs to be considered is the variety of food resources. Honey bees must have access to different type of pollen to cover its essential amino acid needs. It is frequent that beehives in extensive monocultures have access to high amounts of pollen, but since it comes from a single plant species, some of the essential amino acids are usually engaged [29, 30].

Protein supplements have been proven to increase the amount of brood in the beehives, the storage of pollen and honey reserves, and reduce the winter mortality [31, 32]. At individual point, an increase in the lifespan of the bees, the corporal protein concentration, gene expression of vitellogenin, and the size of hypopharyngeal gland of the nurse bees have been observed with protein supplementation [27, 31, 33]. Moreover, sanitary benefits have been described such a reduction of *V. destructor* infestation levels or the increase of gene expression of some antimicrobial peptides such as defensing-1 or abaecin [31].

Other parameters of the beehives, mainly sanitary status, must be kept in mind to improve the production and winter survival of the beehives, as well as the sustainability of this sector. Pathogenic agents such as *V. destructor* and *N. ceranae* are described as some of the main causes of mortality in beehives [10, 13, 20]. The lack of treatments for *N. ceranae*, or the decrease of the efficacy of conventional miticides, due to the appearance of resistant populations of mites, has become increasingly difficult for the management of these diseases [34–39].

Some products have been investigated as feed supplements to improve the sanitary status of the hives. Bioactive compounds, mainly probiotics and postbiotics, have beneficial effects on the natural gut microbiome of the bees and their immune system [40]. In this way, some studies have shown promising results in which bioactive compounds have increased the egg laying of the queen and the storage of pollen and honey, and decreased the loads of *N. ceranae* and *V. destructor* in the beehives [41–44]. However, no studies have evaluated the potential combined effect of postbiotics and protein supplements in honey beehives.

The aim of this study is to evaluate the effect of combination of two different feed supplements, protein and postbiotics, on the strength and sanitary status of the beehives, at two different crucial moments in beekeeping, autumn or pre-wintering period, and the beginning of the spring.

## Material and Methods

### Elaboration of Products

Two types of supplement feed were elaborated. Firstly, a postbiotic product was prepared by the culture of lactic-acid bacteria in liquid MRS culture media (Scharlab®, Barcelona, Spain), isolated from honey bees and larvae samples obtained in apiaries from traditional beekeeping regions from Extremadura, and selected based on the results of previous studies [45, 46]. For this, one colony from a pure culture of each bacterial species was inoculated in 1L of sterile liquid MRS and was culture at 37 °C for 48 h. The concentrations of the cultures were measured by culturing of serial dilutions in sterile solid culture media MRS (Scharlab®, Barcelona, Spain), and they were adjusted to a final concentration of 10^9^ CFU/mL, diluted with sterile MRS. This concentration was determined based on results of previous studies [42, 43, 47, 48]. Lately, they were inactivated by heat at 80 °C for 2 h, and all the growths were mixed in the same proportion for the different bacteria to obtain the final postbiotic product. The absence of live bacteria was checked by culturing the inactivated growths in solid MRS.

Syrup with 1:1 proportion of sucrose was made such as a carrier to feed the beehives with the supplements. This syrup was added with 10 mL of postbiotic product and 5 mL of a commercial liquid protein supplement (Promobee®, Dadelos Apícola, Benifayó, Spain) to feed the beehives. Finally, sucrose syrup without supplements was elaborated to feed the control beehives.

### Experimental Design

This study was divided in two different experiences, one in autumn, between October and November of 2023, and the other in spring of 2024, between March and April. In both experiments, two groups of hives (Layens type) were established, one group supplemented with postbiotic product and the protein supplement, and the other group such as control, which only received the syrup of sucrose. The beehives were distributed in four different commercial apiaries, two for autumn experience and other two for the spring one. The beehives were distributed homogenously between supplemented and control group based on their strength, 38 per group in autumn experience and 23 per group in spring experience, in order to all the beehives were subject the same vegetation, available of food resources, and climate conditions, but in two different apiaries or scenarios to ensure that the results were due to the type of supplementation, as it was described in previous studies [31, 42].

**Fig. 1:**
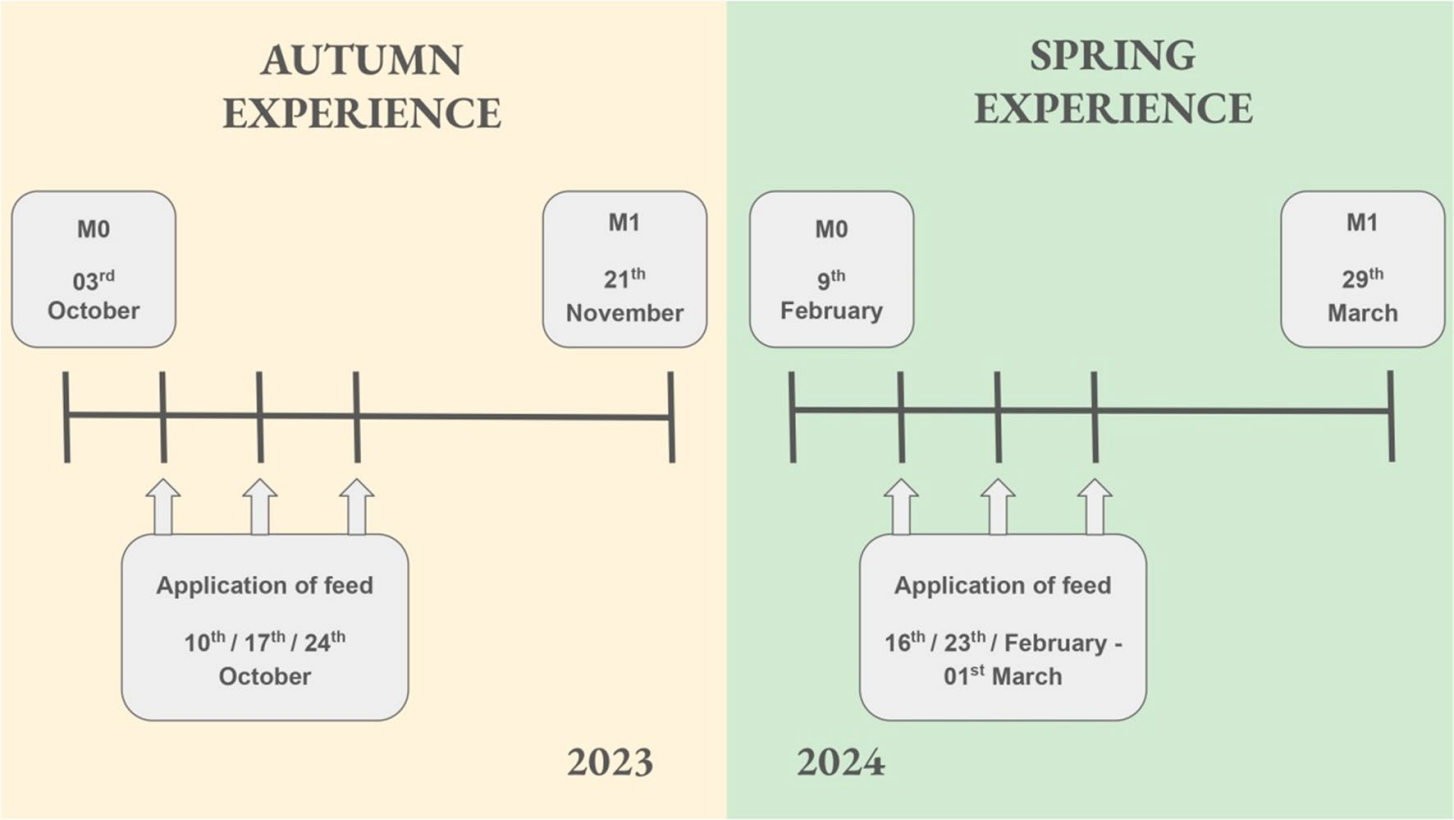
Schedule of experimental design of both experiences

Two monitoring based on the assessment of strength and sanitary status of the beehives were carried out for each experience. Monitoring 0 (M0) was carried out at the beginning of the experience (October 3rd, 2023/9th February, 2024), serving to control the initial status of the beehives and to generate homogeneous groups for each experience. On the other hand, monitoring 1 (M1) was conducted at the end of the experience (21th November, 2023/29th March, 2024), 1 month later than the last application of supplementary feed, serving to assess the effect of feed supplements. Supplementation was performed weekly, in vertical troughs previously installed in the beehives, with a total of three applications, the first one being 1 week after M0. One liter of supplementary feed was administered at each beehive and application. No treatments against *V. destructor* were applied to beehives during the experiments (Fig. 1).

### Monitoring of Beehive Strength

The strength of the beehives was measured in each monitoring of both experiences. For that, the number of adult bees, the amount of brood, and the reserves of pollen and honey were estimated, as was described previously [49]. Briefly, these parameters were estimated by calculating the percentage of the surface of each frame occupied by each one. This percentage was used to estimate the number of adult bees and the area (dm^2^) occupied by brood, pollen, or honey, using established conversion factors. The estimations were performed at the same time zone to reduce the variability among the monitoring.

Moreover, the viability of the capped brood was measured. Thus, the number of empty cells per 100 cells was determined in an area occupied exclusively by capped brood to calculate the percentage of brood survival. This measurement was conducted by triplicate in three different frames and the mean of percentage of brood survival was obtained for each beehive. This parameter was also categorized as “High survival,” when it raised more than 90%; “Medium survival,” when it was between 70 and 90%; and “Low survival,” when it was less than 70%.

Finally, mortality of the beehives of the study was recorded at M1 in both experiences.

### Diseases Diagnosis

On the one hand, approximately 200 nurse bees per beehive from frames with brood were collected and taken to laboratory, where they were stored at − 20 °C to carry out a subsequent diagnosis of phoretic *V. destructor* infestation levels. For that, the bees, once thawed, were immerse in a 5% ethanol solution and were shaken for 5 min. Lately, they were passed through a sieve to separate bees and mites, and both were counted, obtaining the percentage of parasitization or number of mites per 100 adult bees [50].

Moreover, in order to assess the infestation levels of *V. destructor* inside the brood cells, two fragments of approximately 100 capped brood cells were extracted from frames with capped brood in both sides. The number of brood cells was recorded and the pupae and *V. destructor* mites were extracted from cells and counted (adult females/males, and immature mites), obtaining the level of infestation in brood or number of mites per 100 capped brood cells. This estimation was only realized in spring experience, due to the low amount of brood in autumn and in order to avoid damaging the beehives just before winter.

On the other hand, around 30 forager bees per beehive were sampled from frames with honey/pollen and without brood to *N. ceranae* diagnosis purpose, and they were taken to refrigeration at − 20 °C until their utilization. The gut of 20 of these bees were extracted and homogenized in 5 mL of DNAase-free water for 5 min in a homogenizer (Smasher™, bioMérieux, Lyos, France). Subsequently, DNA extraction was carried out using the NukEx Complete Mag RNA/DNA® (Gerbion GmbH & Co. KG, Germany) and a KingFisher™ Flex, Thermo Fisher™ Scientific Inc. The DNA concentration of each sample was measured using a NanoDrop™ 2000 (Thermo Fisher™ Scientific Inc., Waltham, USA), and quantitative PCR was performed, as was previously described [51]. Briefly, for each reaction, 10 μL of Premix Ex Taq™ (2x) (TaKaRa Bio, Kusatu, Japan); 0.2 μM of each primer and ROX Reference Dye; 0.8 μL of Probe; and 2 μL of DNA sample (5 ng of DNA) were taken in a total volume of 20 μL PCR reaction mixture. Cycling parameters of PCR consisted of an initial denaturation at 95 °C for 30 s, followed by 40 cycles of 95 °C for 5 s, and 60 °C for 34 s. Duplicate reactions were performed for template samples, standards, and non-template controls. The number of DNA copies present in each sample was estimated based on standard curve calculated using thermocycler-specific software (Applied Biosystems 7300, Thermo Fisher Scientific Inc., Waltham, USA) and expressed as the number of copies of *N. ceranae* DNA per ng of sample DNA.

### Data Analysis

The percentage of beehives mortality through both experiences, autumn and spring, and categorical brood survival variables were compared among the groups using proportion comparison tests (Chi Square Test (X) or Fisher Exact Test (F)). Dead beehives at the end of the experiences were considered for strength comparisons, but excluded for continuous brood survival variable, *V. destructor* and *N. ceranae* comparisons, due to absence of these parameters data. Strength and sanitary parameters were compared with parametric mean comparison tests (*T*-Student test (T) or paired *T*-Student (T)), among study groups, and between M0 and M1 to analyze the evolution of each parameter in each group, when the variables met the requirements to use them. Otherwise, non-parametric tests were used (Kruskal–Wallis test (K) or paired Wilcoxon test (V)). Statistical analyses were performed using R v4.1.4 software. Differences were considered statistically significant when it was less than 0.05, and *p* values between 0.05 and 0.1 were considered marginally significant.

## Results

Mortality rates were 13.89% (five beehives) in control group and 15.79% (six beehives) in supplemented group in autumn experience (*X* = 6.77E-31; *p* = 1). In spring experience, mortality rates were 4.35% in control (1 beehive) and 0% in supplemented group (F = 0; *p* = 1). No statistical differences were detected among groups in both experiences.

Table 1 lists the mean of strength parameters for both groups and experiences at M0 and M1. In autumn, strength parameters did not show statistical differences among the study groups. The amount of brood decreased significantly in control (K = 6.5; *p* = 0.011) and supplemented group (K = 10.23; *p* = 0.001), in a same way. The remaining parameters continued stable trough the experiment in both groups.

**Table 1:** Mean of strength parameters of the beehives by groups (control (C) and supplemented (S)) and monitoring, in autumn and spring. Adult bees are expressed in terms of the number of adult bees; brood, pollen reserves, and honey reserves such as area (dm^2^) occupied by each one; and brood survival such as percentage of brood survival. Values marked with * show statistical differences (or marginal differences) among groups, and values marked with ^♦^ show statistical differences (or marginal differences) between M0 and M1 within the same group

Group	Adult bees		Brood		Brood survival		Pollen reserves		Honey reserves	
	M0	M1	M0	M1	M0	M1	M0	M1	M0	M1
Autumn										
C	8331	7779	35.34^♦^	17.84^♦^	90.36	91.32	20.34	16.09	73.89	60.74
S	7255	6313	33.04^♦^	15.08^♦^	89.95	91.79	23.67	16.81	78.15	66.25
Spring										
C	4831^♦^	8609^♦^	32.58^♦^	51.59^♦^	91.47*^♦^	84.61^♦^	24.86^♦^	33.06^♦^	29.2	36.79
S	5515^♦^	9812^♦^	35.91^♦^	46^♦^	85.19*	83.47	25.39^♦^	35.08^♦^	36.35^♦^	46.34^♦^

In spring experience, the number of adult bees in the beehive increased significantly between M0 and M1 in control (T =  − 3.74; *p* = 5.6e-04) and supplemented groups (K = 15.13; *p* = 1E-04). Similarly, the amount of brood increased through the experience in both groups (control T =  − 2.54; *p* = 0.016; supplemented K = 3.09; *p* = 0.079), as well as the pollen reserves, which were improved among the monitoring in control (K = 3.09; *p* = 0.079) and supplemented groups (T =  − 2.25, *p* = 0.03). None of these parameters showed statistically significant differences among groups.

**Fig. 2:**
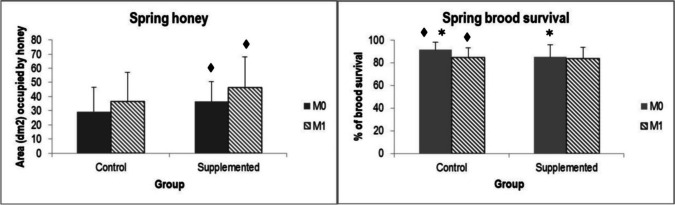
Mean of reserves of honey and brood survival in spring, by groups and monitoring. Values marked with * show statistical (or marginal) differences among groups, and values marked with ♦ show statistical (or marginal) differences between M0 and M1 in the same group

Regarding honey reserves, no significant differences were detected among groups. However, the supplemented group experienced a marginally significant increase between M0 and M1 (K = 2.98, *p* = 0.085), while control did not show statistical differences (Fig. 2).

**Fig. 3:**
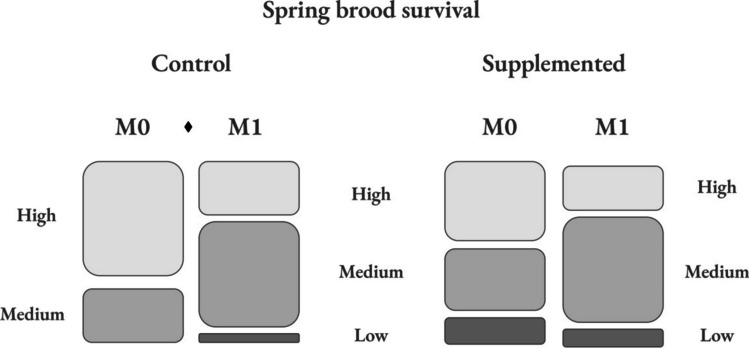
Brood survival proportions were classified in high (> 90%), medium (70–90%), and low (< 70%) by group and monitoring. The symbol ♦ indicates statistical differences between M0 and M1

Finally, with regard to strength parameters, Table 2 shows categorical brood survival by group and monitoring in autumn and spring. In autumn, the brood survival and categorical brood survival remained stable through the experiences in both groups, and statistical differences were not detected among groups. However, in spring experience, although the control group began with a higher brood survival rate than supplemented group (K = 3.55; *p* = 0.06), beehives of this group decreased both brood survival (K = 6.61; *p* = 0.01) (Fig. 2) and categorical brood survival between M0 and M1 (F = 5.58; *p* = 0.05), whereas the supplemented group maintained these parameters at similar values (Fig. 3).

**Table 2:** Percentage of beehives with high (> 90%), medium (70–90%), and low (< 70%) brood survival by group (control (C) and supplemented (S)) and monitoring in both experiences. Values marked with ♦ show statistical differences (or marginal differences) between M0 and M1 in the same group

Group	M0			M1		
	High	Medium	Low	High	Medium	Low
Autumn brood survival						
C	65.52	34.48	0	72.41	27.59	0
S	65.39	26.92	7.69	69.23	26.92	3.85
Spring brood survival						
C	68.42^♦^	31.58^♦^	0^♦^	31.58^♦^	63.16^♦^	5.26^♦^
S	47.37	36.84	15.79	26.32	63.16	10.53

Regarding diseases diagnosis, Table 3 shows mean values of *V. destructor* and *N. ceranae* infestation levels by groups and monitoring in both experiences. Infestation levels of phoretic *V. destructor* significantly increase through the autumn experience in control group (K = 5.1; *p* = 0.024), while in supplemented groups, it remained in similar values, showing control in a higher infestation level than supplemented at the end of the experiment (K = 2.91; *p* = 0.088). In spring, no significant differences were detected among groups or monitoring.

**Table 3:** Mean of diseases diagnosis parameters of the beehives by groups (control (C) and supplemented (S)) and monitoring, in autumn and spring experiences. Phoretic *V. destructor* is expressed such as number of mites/100 bees; brood *V. destructor* such as number of mites/100 capped brood cells; and *N. ceranae* such as number of copies of *N. ceranae* DNA/ng sample DNA. Values marked with * show statistical differences (or marginal differences) among groups, and values marked with ^♦^ show statistical differences (or marginal differences) between M0 and M1 in the same group

Group	Phoretic *V. destructor*		Brood* V. destructor*		*N. ceranae*	
	M0	M1	M0	M1	M0	M1
Autumn						
C	1.039^♦^	2.439*^♦^	-	-	57,659.61^♦^	79,421.61^♦^
S	1.123	1.351*	-	-	32,847.24^♦^	27,120.1^♦^
Spring						
C	0.439	0.981	0.54*	1.952	57,539.7	47,200.78
S	0.856	1.327	1.647*	1.956	38,516.4	53,938.19

**Fig. 4:**
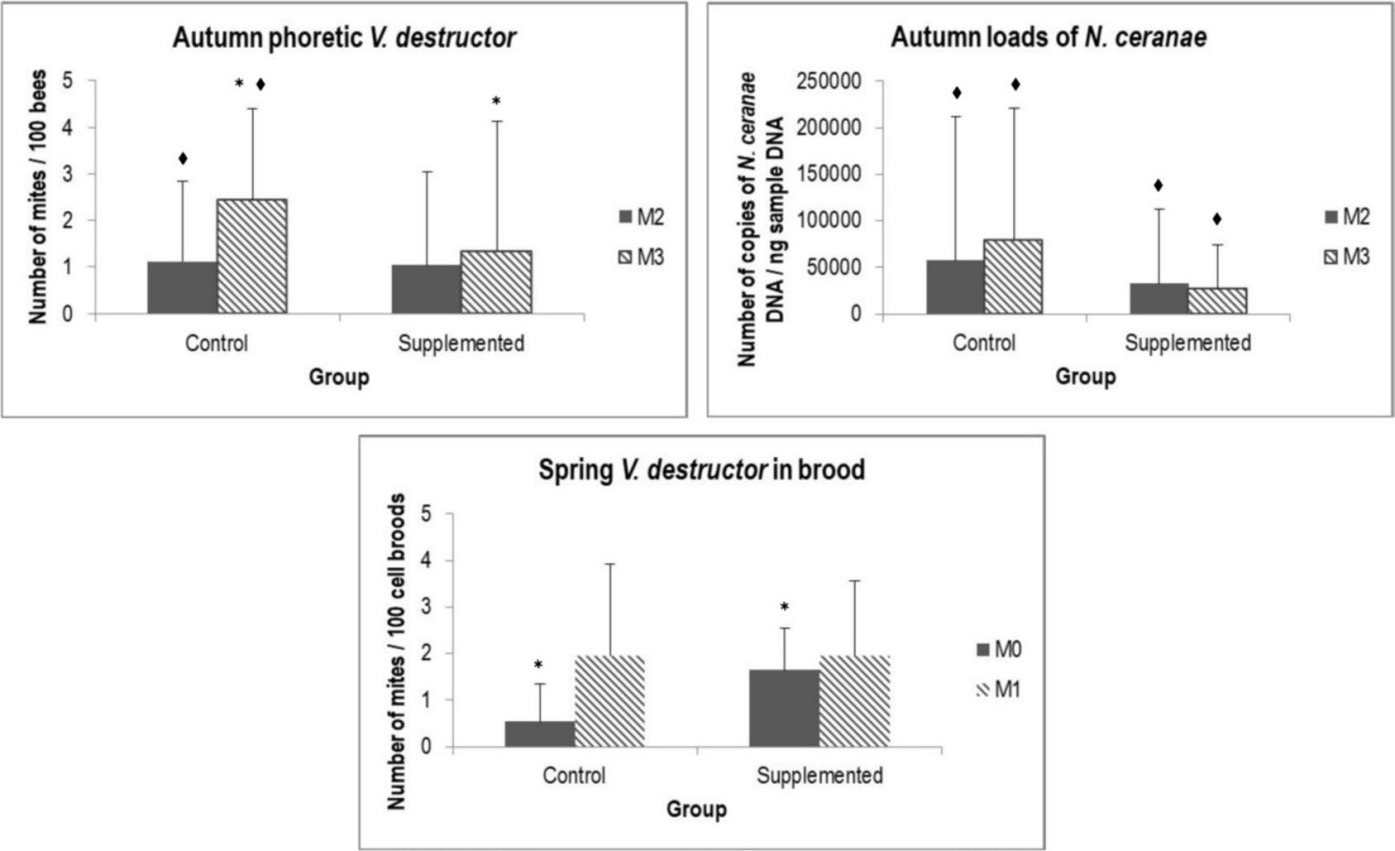
Mean of phoretic *V. destructor*, loads of *N. ceranae* (autumn), and *V. destructor* in brood (spring) by groups and monitoring. Values marked with * show statistical (or marginal) differences among groups, and values marked with ♦ show statistical (or marginal) differences between M0 and M1 in the same group

Regarding *V. destructor* in brood, this parameter was only estimated in spring. In this case, although the supplemented group began the experience with a significantly high infestation level than control (K = 2.93; *p* = 0.087), both groups reach similar values at M1, without significant differences among them (Fig. 4).

Finally, the loads of *N. ceranae* in autumn did not show statistical differences among groups, but a significant increase of this pathogen between M0 and M1 was detected in control (K = 4.25; *p* = 0.039), while supplemented group significantly decrease its load through the experience (K = 3.32; *p* = 0.068) (Fig. 4). In spring, no statistical differences were observed among groups or monitoring. None of the studied parameters showed differences in the study groups among apiaries, and the trends were similar in both locations.

## Discussion

The results of this study showed for first time that feed supplementation of beehives with protein and postbiotics combination improved some beehives parameters related to the strength and sanitary status. Previous studies with single protein supplementation in summer showed an increase in brood and a decrease of *V. destructor* levels [31]. On the other hand, the results obtained in previous studies with single postbiotic supplements in summer showed higher quantity of brood and higher number of bees, as well as a reduction in *N. ceranae* levels [42]. For this reason, we tested the combination of both products, which joined their both beneficial effects manifesting differently depending on the season of administration. Notable improvements from the combination were observed such as the increase of honey yield in spring.

Firstly, in the autumn experience, the improvements obtained in the beehives after feed supplementation were mainly related with sanitary parameters. The infestation levels of phoretic *V. destructor* remained stable through the experience in supplemented beehives while in control group they increased by more than 100%. Both protein and postbiotic supplements have previously shown promising effects against *V. destructor*, limiting the increase of population of the mite in the beehives [31, 41]. It would have been advisable to data on the levels of *V. destructor* in brood as well, but due to the dates in which the study was conducted, it was decided to not to take these samples so as not to damage the beehives and not to put them at risk. In similar way, the infestation levels of *N. ceranae* were reduced in supplemented hives, while increased in control group. These results have been previously described for postbiotics such as feed supplement in beehives [42, 52].

The protein contribution has been able to improve the nutritional status of the bees that have been described such as key parameter to face the pathogens, contributing the enhancement of the immune system by improving the synthesis of antimicrobial peptides as well as the hygienic behavior of the bees, which are capable to easily remove the mites [53–56]. The effects of the postbiotics against bee pathogens may be attributed to multiple factors. In the first place, the interaction and enhancement of the immune system and metabolites contained in postbiotics have been largely described [40, 45, 57]. Moreover, the organic acids and other metabolites such as surfactin, which are produced by lactic-acid bacteria, seem to play a key role against *N. ceranae*, damaging the spore external structure [58, 59]. Moreover, it is possible that the combined use of protein and postbiotics had a synergistic effect enhancing the honey bee immune system and reducing pathogens infestation levels, but the mechanism of this possible synergistic relationship has not been evaluated in this study. These mechanisms of possible synergy and the composition of both products, mainly the postbiotic, under the conditions inside the beehive until their consumption will be included in future studies to a better comprehension of how they interact among them and with the honeybees. Moreover, the relationship among both pathogens have been described previously, so that *N. ceranae* dynamics depend mainly on seasonality and climatic conditions, but also on the levels of parasitization by *V. destructor*, so that greater parasitization by the mite favors higher levels of infestation by *N. ceranae* [60]. Thus, the decreased of *V. destructor* infestation levels by the supplements could also have played a part in the reduction of *N. ceranae* loads.

Keeping in mind the high winter mortality rates of the beehives in the last years [5–8], and that both *V. destructor* and *N. ceranae* have been described as two of the main causes of this current problem [10, 13, 20], the decrease of their infestation levels by feed supplementation with combination of protein and postbiotics before the cold season could be a promising tool to increase the survival of the beehives and reduce the mortality rates.

Regarding the strength of the beehives, at the time that study is carried out, the colonies of *Apis mellifera* are preparing to remain confined in the hive through the cold season. Winter is a period of scarcity for the honey bees, without sources of pollen and nectar available in the field, and with weather conditions of cold, rain, and wind that are very hard for them, making it difficult for them to leave the beehive for weeks or even months. In this scenario, the beehives must reduce their population and their nutritional needs at minimum levels to survive until the spring. For this reason, at the end of the autumn, the queen bee begins to reduce the egg laying, the bee population reduces, and bees start to use the reserves of pollen and honey collected during the spring, summer, and early autumn. Thus, both study groups reduced their bee population, amount of brood, and food reserves through autumn experience, as a way of adapting to approaching cold season [61, 62].

Some studies have proved that application of protein supplements in the beehives in late autumn/early winter increased the amount of brood, and reserves of pollen and honey, but these effects were recorded after winter, during the next spring after supplementation, when the colony began to grow and weather conditions were again favorable [32, 63]. Maybe in the same way, in our study, some of these improvements in the spring dynamic population of the supplemented beehives could have been recorded, but the last monitoring in the autumn experience was at the end of November, and the evolution of the strength of the colonies was similar in both control and supplemented groups through the experience. The same occurs with supplementation with postbiotics, which have been shown to increase the egg laying of the queen and brood levels, but when they have been applied in spring or summer, when the bee population is growing [42, 43, 52].

On the other hand, in spring experience, the feed supplementation of the beehives showed an improvement in some parameters such as the brood survival, the honey yield, and *V. destructor* infestation levels in brood. The survival of the brood through the experience remained similar values in supplemented hives, while in control group, it decreased, despite it began with a higher brood survival. Most of the studies previously carried out to evaluate protein or postbiotics supplements focused on the amount of brood, recording an increase of this parameter with supplements [27, 31, 32, 52, 63, 64], but they did not evaluate its survival. The higher initial brood survival and its decreased towards the end of the experiment could be the reason of no differences detected among groups for the number of adult bees or amount of brood, since the consequences of the decline of brood survival in control group could be remarkable in a longer period, necessary to the bees for complete the growth cycle.

This reduction in brood survival was probably due to the increase of *V. destructor* in brood. While the infestation level of the mite in brood was similar through the experience in supplemented beehives, in the control group increased it by more than 250%. A reduction in development and the weight of the pupae has been described by *V. destructor* infestation, regardless of whether individual pupae were parasitized or not, so the quality of brood care is a likely colony-level effect [65, 66]. Phoretic *V. destructor* had a similar evolution in both groups trough the experiences. It is possible that over a longer period its increase in brood would have been reflected in phoretic *V. destructor* of control group. Thus, protein and postbiotic supplementation maintained the *V. destructor* in brood and brood survival, while the mite infestation notably increased and brood survival decreased in beehives of control group, in line with what is described in literature for the effect of these types of supplements in *V. destructor* [31, 52].

Regarding *N. ceranae*, while in autumn, the supplementation of the beehives showed a reduction of its infestation levels; in spring experience, no differences were detected between groups or monitoring. This may be due just for the seasonality of the experiences. In spring, this pathogen is at the moment of greatest reproduction and expansion in the beehives, in an exponential way [67], so the effect of supplements may be less noticeable.

Lastly, other parameter affected by supplements was the honey reserves, which significantly increased through the spring experience in supplemented beehives. Some parameters such as the improvement of fly ability, thorax muscle mass, weight, or wing and body size have been demonstrated when optimal nutrition of the bees is ensured [68–70]. This may be the reason of the increase of honey yield in supplemented beehives. Although the bee population was similar in both groups, this improvement in the functionality of the worker bees could cause a higher foraging activity, generating larger reserves of honey in the beehives. Improvements in foraging and storage of nectar and pollen have been documented in some protein supplementary diets previously [32, 71]. In the present study, significant differences were detected only in honey reserves in supplemented group. Despite the absence of statistical differences, a similar trend was observed towards a greater increase of pollen reserves in supplemented hives.

To the best of our knowledge, this is the first report of beehives supplemented with a combination of postbiotics and protein under field conditions. The improvements obtained in spring experience, such as the increased of brood survival and honey yield, and the limitation of *V. destructor* expansion in the supplemented beehives, could lead to stronger beehives with a higher ability to rear healthy brood and collect reserves for the beehive in the season with more food available in the field. This increase in honey production is a crucial parameter for beekeepers, making their colonies more productive and generating greater benefits.

On the other hand, these supplements used in autumn could be an important tool to reduce the mortality rates of the beehives and to ensure a good survival rate through the winter, by improving their sanitary status.

## Data Availability

No datasets were generated or analysed during the current study.
